# A Visco-Hyperelastic Constitutive Model to Characterize the Stress-Softening Behavior of Ethylene Propylene Diene Monomer Rubber

**DOI:** 10.3390/polym15163388

**Published:** 2023-08-12

**Authors:** Xiu Liu, Chen Liu, Dingxiang Zhu, Jianguo Lin

**Affiliations:** 1 School of Mechanical Engineering and Mechanics, Xiangtan University, Xiangtan 411105, China; dxzhu98@gmail.com; 2 School of Civil Engineering, Xiangtan University, Xiangtan 411105, China; 202121572201@smail.xtu.edu.cn; 3 School of Materials Science and Engineering, Xiangtan University, Xiangtan 411105, China

**Keywords:** stress-softening effect, visco-hyperelastic constitutive model, biaxial stretching, pseudo-elastic model, stress relaxation

## Abstract

Uniaxial and biaxial cyclic tensile tests and stress relaxation tests were performed on the ethylene propylene diene monomer (EPDM) material to investigate its stress-softening effect. The experimental results reveal that the EPDM material presents a significant Mullins effect during the cyclic stretching processes. Furthermore, it is found that the deformation of the EPDM material does not return to zero simultaneously with the stress, due to the viscoelasticity of the EPDM material. Therefore, this study combines pseudo-elasticity theory and viscoelastic theory to propose a visco-hyperelastic constitutive model. The proposed model is used to fit and analyze the uniaxial and biaxial cyclic test results of EPDM and a comparison is conducted with the corresponding hyper-elastic constitutive model. The results show that the proposed model is in good agreement with the experimental data and superior to the hyper-elastic constitutive model, especially when it comes to the stress-softening unloading process. This work is conducive to accurately characterizing the stress-softening behavior of rubber-like materials at large deformation and can provide some theoretical guidance for their widespread application in industry.

## 1. Introduction

Rubber materials are extensively used in fields such as aerospace, shock absorption and sealing, and flexible electronic devices due to their excellent elasticity, flexibility, wear resistance, and electric insulation [1]. As a type of polymer material, they also have viscoelasticity, so the strength and fatigue life of rubber components decreases with increasing temperature and frequency [2]. Ethylene propylene diene monomer (EPDM) rubber contains saturated hydrocarbons in its main chain and is chemically stable with good thermal insulation, aging resistance, wear resistance, and resistance to chemical media, etc. It plays an important role in various engineering fields, such as tunnel waterproofing [3], electromagnetic shielding [4], thermal insulation [5], etc. Consequently, it is of terrific practical significance to study the complex behavior of EPDM rubber due to its great potential for advancement.

The Mullins effect refers to a stress softening phenomenon where the loading curve is frequently higher than the unloading curve and the loading-unloading stress–strain curve of rubber material forms a hysteresis loop [6]. The Mullins effect has been studied for more than seventy years, but it is still regarded as a main challenge in the study of the complex mechanical behavior of rubber-like materials. In order to better understand the stress softening caused by the Mullins effect, Julie et al. [7] summarized several physical explanations, ranging from chain breakage at the interface between the rubber and the filler to the sliding of molecules and the formation of more complex composite structures.

In recent years, there have been many investigations into the phenomenon of stress softening. For example, Nicolas et al. [8] studied the thermomechanical coupling of rubber Mullins damage and demonstrated that Mullins damage is essentially related to the activation of the dissipative cavitation mechanism. Li et al. [9] investigated the specific rules of the Mullins effect in rubber nanocomposites. Morovati et al. [10] explored fatigue-induced stress-softening in cross-linked multi-network elastomers, which is expected to offer insights into the complexity of constitutive behavior in multi-network elastomers. Bahrololoumi et al. [11] developed a new micro-mechanical model to predict the non-linear behavior of rubber-like materials such as the idealized Mullins effect and permanent set during hydrolytic aging.

For the complex Mullins effect, it is evident that no existing model can accurately and efficiently capture all the characteristic stress responses of an elastomer’s stress softening, rate and temperature dependence, strong nonlinearity, etc., in a thermodynamically consistent and numerically robust manner. As a result, many models only consider the idealized stress-softening phenomenon without taking into account additional impacts, such as permanent set [12,13]. Numerous hyper-elastic constitutive models have been proposed by researchers in an effort to describe the mechanical behavior of stress softening. These models can be broadly divided into two categories: The first group is based on micro statistical mechanics and assumes that the material is made up of randomly distributed molecular chains. This type of model includes the Kuhn–Grun three-chain model, the Flory four-chain model, and the Arruda–Boyce eight-chain model [14], etc. The second category is the phenomenological model, which applies the method of continuum mechanics and assumes that the elastic potential is made up of invariants constructed by the elongation ratio or the deformation tension. These models, such as the Mooney–Rivlin model [15], the Ogden model [16], and the Yeoh model [17], identify the various parameters in the elastic potential based on experimental data. In order to explain the Mullins effect, Ogden et al. [18] proposed a simple phenomenological model, the pseudo-elasticity theory model, which is simpler than the stress-softening model proposed by Mullins, to more precisely describe the mechanical behavior of stress softening. This model is based on the hyper-elastic constitutive models mentioned above. In addition to the traditional hyper-elastic constitutive models, some new hyper-elastic constitutive models have also been developed recently. For example, Yao et al. [19] proposed a constitutive model to explain the cyclic response of hyper-elastic NiTi shape memory alloy, and Nguyen et al. [20] put forward an analytical model for the addition and removal cycles of hyper-elastic memory alloy beam. To model the hyper-elastic fracture and fatigue behavior of memory alloys, Simoes et al. [21] offered a native model; Viet et al. [22] presented a new hyper-elastic cyclic model for the behavior of large refractive index hyper-elastic shape memory alloy coil springs.

Although the aforementioned hyper-elastic models have been well established, they are still not good enough to describe the stress-softening effect of rubber-like materials. Particularly, the loading–unloading curve depicted by these models always forms a closed loop, which is inconsistent with the facts. This is because in addition to dramatic hyper-elastic deformation, rubber-like materials also show complex viscoelastic deformation that prevents the hysteresis loop of stress softening from opening. As a consequence, viscoelasticity must be considered in order to fully describe the mechanical behavior of stress softening. There are two types of viscoelasticity: linear and non-linear. The former is generally described by the Maxwell model, Kelvin model and three-parameter solid model, etc. However, the mechanical properties of rubber materials present more complicated nonlinearity, which is challenging to explain with straightforward linear viscoelastic models.

Some nonlinear viscoelastic models have been proposed by researchers, such as BKZ theory [23], the Christensen equation [24], and generalized line integral theory [25]. In addition to the classical nonlinear visco-elastic constitutive models, some new nonlinear viscoelastic constitutive models have also been proposed recently, such as the free volume-based nonlinear viscoelastic model for polyurea over a wide range of strain rates and temperatures [26], the nonlinear viscoelastic model of mucociliary clearance [27], the nonlinear viscoelastic model of adhesive failure for polyacrylate pressure-sensitive adhesives [28], and the nonlinear uniaxial stress-strain constitutive model for viscoelastic membrane materials [29]. In view of the above problems, researchers developed a kind of constitutive model that could reflect both the hyper-elasticity and the viscoelasticity of rubber materials, namely, the visco-hyperelastic constitutive model. For example, Huang et al. [30] developed a visco-hyperelastic constitutive model of a porous structural material, poly-siloxane silica, at large strain rates, and Tan et al. [31] developed a transverse isotropic visco-hyperelastic constitutive model for short-fiber reinforced EPDM rubber. Although there are currently numerous constitutive models for rubber materials, most of the existing models are hyper-elastic models, which are usually only used to describe the super elasticity of the rubber-like material and do not involve its viscoelasticity. The existing visco-elastic models mainly focus on the uniaxial mechanical behavior in the loading stage.

It is challenging to develop an accurate and comprehensive constitutive model of the EPDM rubber under finite deformation because the EPDM rubber has obvious nonlinear properties and is affected by environmental factors such as loading rates, loading histories and external temperature. In addition, although the real rubber components are usually in complex stress states, previous research has mostly concentrated on the unidirectional stress state, but less on the bidirectional or tri-directional stress state. Therefore, it is of great scientific interest to study the visco-hyperelastic mechanical behavior of EPDM rubber under the biaxial stress state. Numerous techniques have been developed for the study of the biaxial stress state. The most popular are uniaxial compression in place of the biaxial tensile testing method [32], the bulge tensile testing method [33], the pressure vessel biaxial tensile testing method [34], and the cross-shaped specimen biaxial tensile testing method [35]. The last method will be employed for the biaxial stretching tests in this work, due to its advantages of widespread applications, efficient test control, and straightforward equipment design.

This research attempts to develop a visco-hyperelastic constitutive model and applies it to characterize the uniaxial and biaxial stress-softening behavior of EPDM rubber, which has great potential in pillar industries of national production. The proposed model is constructed by combining a hyper-elastic element and a viscoelastic element. Then, the model parameters of the proposed model were determined according to uniaxial and equal biaxial stress relaxation tests and cyclic tensile tests of EPDM rubber. Finally, the proposed visco-hyperelastic constitutive model was compared with the corresponding hyper-elastic model to verify the effectiveness of the model in this research. Compared with the existing models, the visco-hyperelastic constitutive model proposed in the current study takes into account the nonlinear viscoelasticity and super elasticity of the rubber-like material. It can accurately characterize the stress-softening behavior of rubber-like materials during loading and unloading processes. Moreover, the proposed model extends the uniaxial stress state to the biaxial stress state, which is more closely related to the actual stress state of rubber-like materials in practical engineering. The proposed model in this work is crucial for the accurate description of the material’s stress-softening mechanical behavior, especially under the complex stress states, which can provide effective support for characterizing the mechanic behaviors of rubber-like materials at large deformations and provide certain theoretical guidance for the design and application of rubber-like materials.

## 2. Construction of Visco-Hyperelastic Constitutive Model

**Figure 1:**
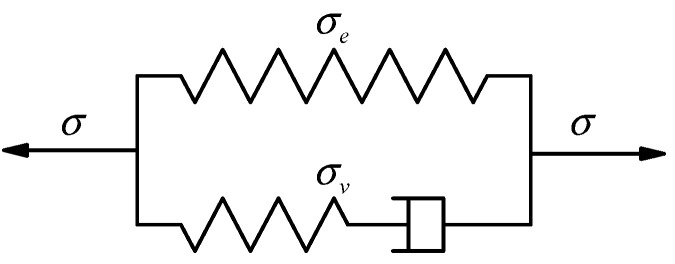
The parallel visco-hyperelastic constitutive model.

In this work, a visco-hyperelastic constitutive model is constructed, which consists of a hyper-elastic unit and a visco-elastic unit in parallel, as shown in Figure 1.

The visco-hyperelastic constitutive model can be expressed as [36]:

(1) $$\sigma =\sigma_{e}+\sigma_{v}$$

where σ is the total stress, $\sigma_{e}$ is the time-independent nonlinear hyper-elastic stress, and $\sigma_{v}\;$ is the time-dependent nonlinear viscoelastic stress.

### 2.1. Hyper-Elastic Constitutive Model

Hyper-elastic constitutive models of the rubber material can be broadly classified into two main categories based on the statistical properties of molecular chains [37]: phenomenological models and network models. The key to establishing the phenomenological models is to construct a reasonable strain energy function *W*, which is expressed as a function of deformation tensor invariant $I_{i}(i=1,2,3)$, such as the Mooney–Rivlin model and the Yeoh model, or as a function of the elongation ratio $\lambda_{i}(i=1,2,3)$, such as the Ogden model and the Valanis–Landel model. The Neo-Hooke model is a Gaussian chain network model, while the James–Guth three-chain model, the Flory four-chain model, the full-chain network model and the Arruda–Boyce eight-chain model are non-Gaussian chain network models. In this work, the Yeoh constitutive model and the Ogden constitutive model, commonly used to describe large deformation behavior, are used as the hyper-elastic unit principal structure models.

In the description of rubber materials in the present constitutive model, rubber materials are considered as isotropic incompressible materials, and the principal stretching ratios of uniaxial and equal biaxial tension $\lambda_{i}(i=1,2,3)$ are [37]:

(2) $$\left\{\begin{array}{l} ST:\;\lambda_{1}=\lambda ,\lambda_{2}=\lambda_{3}=\lambda^{-1/2}\\ ET:\lambda_{1}=\lambda_{2}=\lambda ,\lambda_{3}=1/\lambda^{2} \end{array}\right.$$

where *ST* and *ET* denote uniaxial stretching and equal biaxial stretching, respectively.

#### 2.1.1. The Yeoh Hyper-Elastic Constitutive Model

The strain energy function of the Yeoh model can be expressed as [17]:

(3) $$W_{Yeoh}=\sum_{i=1}^{3}C_{i0}{\left(I_{1}-3\right)}^{i}$$

where $I_{1}=\lambda_{1}^{2}+\lambda_{2}^{2}+\lambda_{3}^{2}$ is the first deformation tensor invariant. Substituting Equation (2) into Equation (3), the strain energy functions of the Yeoh model of rubber in both uniaxial and equal biaxial deformation modes can be obtained:

(4) $$\left\{\begin{array}{l} ST:W^{ST}=\sum_{i=1}^{3}C_{i0}{\left(\lambda^{2}+2\lambda^{-1}-3\right)}^{i}\\ ET:W^{ET}=\sum_{i=1}^{3}C_{i0}{\left(2\lambda^{2}+\lambda^{-4}-3\right)}^{i} \end{array}\right.$$

According to the nominal stress P=∂W/∂λ, the nominal stress–stretch ratio relationship of the Yeoh hyper-elastic constitutive model in uniaxial and equal biaxial cases can be derived:

(5) $$\left\{\begin{array}{l} ST:P^{ST}=\sum_{i=1}^{3}2iC_{i0}^{}{\left(\lambda^{2}+2\lambda^{-1}-3\right)}^{i-1}(\lambda -\lambda^{-2})\\ ET:P^{ET}=\sum_{i=1}^{3}2iC_{i0}^{}{\left(2\lambda^{2}+\lambda^{-4}-3\right)}^{i-1}(\lambda -\lambda^{-5}) \end{array}\right.$$

where $C_{i0}$ is the material parameter.

#### 2.1.2. The Ogden Hyper-Elastic Constitutive Model

The strain energy function of the Ogden model is expressed as [16]:

(6) $$W_{Ogden}=\sum_{i=1}^{N}\frac{2\mu_{i}}{\alpha_{i}^{2}}\left(\lambda_{1}^{\alpha_{i}}+\lambda_{2}^{\alpha_{i}}+\lambda_{3}^{\alpha_{i}}-3\right)$$

where $\mu_{i}$ and $\alpha_{i}$ are arbitrary constants. The number of summation terms can be adjusted to fit the test data accurately, so the Ogden model is more flexible. For isotropic incompressible materials, the Ogden model can be taken as N=3 in the main loading path.

Substituting Equation (2) into Equation (6), the strain energy functions of the Ogden model of rubber in both uniaxial and equal biaxial deformation models can be obtained:

(7) $$\left\{\begin{array}{l} ST:W^{ST}=\sum_{i=1}^{3}\frac{2\mu_{i}}{\alpha_{i}^{2}}\left(\lambda^{\alpha_{i}}+2\lambda^{-\frac{\alpha_{i}}{2}}-3\right)\\ ET:W^{ET}=\sum_{i=1}^{3}\frac{2\mu_{i}}{\alpha_{i}^{2}}\left(2\lambda^{\alpha_{i}}+\lambda^{-2\alpha_{i}}-3\right) \end{array}\right.$$

In the same way, according to the nominal stress P=∂W/∂λ, the nominal stress–stretch ratio relationship of the Ogden hyper-elastic constitutive model for uniaxial and equal biaxial cases can be derived:

(8) $$\left\{\begin{array}{c} ST:\;P_{}^{\mathrm{ST}}=\sum_{i=1}^{3}\frac{2\mu_{i}^{}}{\alpha_{i}^{}}\left(\lambda^{\alpha_{i}^{}-1}-\lambda^{-\frac{1}{2}\alpha_{i}^{}-1}\right)\\ \;ET:\;P_{}^{\mathrm{ET}}=\sum_{i=1}^{3}\frac{2\mu_{i}^{}}{\alpha_{i}^{}}\left(\lambda^{\alpha_{i}^{}-1}-\lambda^{-2\alpha_{i}^{}-1}\right) \end{array}\right.$$

### 2.2. Pseudo-Elastic Model

Ogden et al. [18] proposed a simple phenomenological model to explain the Mullins effect based on the incompressible isotropic elastic theory. In the model, the strain energy function is modified by introducing damage parameters, so that the strain energy function is different from the strain energy function on the main loading path from the original state. On this account, this model is described as a pseudo-elastic model whose main loading-unloading cycle involves energy dissipation. The damage function calculates the ability to dissipate, which solely depends on the damage parameters and the starting point of unloading on the main loading path. The model combines the usual form of the strain energy function with two particular forms of modifiable material constants to highlight the qualitative characteristics of the Mullins effect under simple tension and pure shear circumstances. Additionally, the model created by this theory can be used for multi-axial stress-strain states in addition to uniaxial tests. One of the function expressions that can derive the continuous damage parameter η is:

(9) $$\eta =\left\{\begin{array}{l} 1\\ 1-\frac{1}{r}erf[\frac{1}{m}(W_{m}-W(\lambda_{1},\lambda_{2}))] \end{array}\right.\begin{array}{c} Load\\ Unload \end{array}$$

where $W_{m}$ is the strain energy corresponding to the maximum stretch ratio on the loading path; erf⁡(⋅) is the error function; $W\left(\lambda_{1},\lambda_{2}\right)$ is the strain energy function used to describe the original loading path within a specified range; and parameter *r* is a measure of the damage degree relative to the initial state. The larger the value of the parameter *r*, the less likely the continuous damage function η will deviate from the unity, and the less damage will occur. Parameter *m* controls the dependence of damage on the degree of deformation. For smaller values of *m*, small strains cause significant damage and the material response in the small strain region will not be significantly affected by further loading; for larger values of *m*, small strains cause relatively little damage, but the material response will change significantly in the small strain region after subsequent loading.

#### Pseudo-Elastic Model for Uniaxial and Equal Biaxial Cases

For the stress-softening effect of rubber, it is necessary to introduce the continuous damage parameter η to the hyper-elastic material constitutive model.

In the case of *ST*, the principal stress and principal stretch ratio are:

(10) $$\left\{\begin{array}{l} \sigma_{1}=\sigma ,\sigma_{2}=\sigma_{3}=0\\ \lambda_{1}=\lambda ,\lambda_{2}=\lambda_{2}=\lambda^{-1/2} \end{array}\right.$$

In the case of *ET*, the principal stress and principal stretch ratio are:

(11) $$\left\{\begin{array}{l} \sigma_{1}=\sigma_{2}=\sigma ,\sigma_{3}=0\\ \lambda_{1}=\lambda_{2}=\lambda ,\lambda_{3}=\lambda^{-2} \end{array}\right.$$

Substituting Equations (10) and (11) into Equation (9) yields the expression of the corresponding continuous damage function as:

(12) $$\left\{\begin{array}{l} ST:\;\eta^{ST}=\left\{\begin{array}{l} 1\\ 1-\frac{1}{r}erf\left[\frac{1}{m}\left(W_{m}-W\left(\lambda ,\lambda^{-1/2}\right)\right)\right] \end{array}\right.\begin{array}{c} Load\\ Unload \end{array}\\ ET:\;\eta^{ET}=\left\{\begin{array}{l} 1\\ 1-\frac{1}{r}erf\left[\frac{1}{m}\left(W_{m}-W\left(\lambda ,\lambda \right)\right)\right] \end{array}\right.\begin{array}{c} Load\\ Unload \end{array} \end{array}\right.$$

Although the Mullins effect is a distinctive stress-softening property of rubber-like materials, it is frequently overlooked by the hyper-elastic constitutive model of these materials. To account for the Mullins effect in rubber-like materials, a pseudo-elastic constitutive model considering the damage effect can be written as [18]:

(13) $$\sigma_{e}=\eta (\lambda_{1},\lambda_{2})\times P(\lambda_{1},\lambda_{2})$$

where $\sigma_{e}$ is the time-independent nonlinear hyper-elastic stress, $\eta \left(\lambda_{1},\lambda_{2}\right)$ is the continuous damage function of the pseudo-elastic model, and $P\left(\lambda_{1},\lambda_{2}\right)$ is the constitutive model of the hyper-elastic part. Then, the Yeoh pseudo-elastic constitutive model for uniaxial and equal biaxial cases can be obtained by substituting Equations (4), (5) and (12) into Equation (13).

In the case of *ST*, we can obtain:

(14) $$\sigma_{e}^{ST}=\left\{\begin{array}{lll} \sum_{i=1}^{3}2iC_{i0}^{}{\left(\lambda^{2}+2\lambda^{-1}-3\right)}^{i-1}(\lambda -\lambda^{-2}) & & Load\\ \left\{1-\frac{1}{r^{}}erf\left[\frac{1}{m}\left(W_{m}-\sum_{i=1}^{3}C_{i0}{\left(\lambda^{2}+2\lambda^{-1}-3\right)}^{i}\right)\right]\right\}\times & & \\ & & Unload\\ \sum_{i=1}^{3}2iC_{i0}^{}{\left(\lambda^{2}+2\lambda^{-1}-3\right)}^{i-1}(\lambda -\lambda^{-2}) & & \end{array}\right.$$

In the case of *ET*, we can obtain:

(15) $$\sigma_{e}^{ET}=\left\{\begin{array}{lll} \sum_{i=1}^{3}2iC_{i0}^{}{\left(2\lambda^{2}+\lambda^{-4}-3\right)}^{i-1}(\lambda -\lambda^{-5}) & & Load\\ \left\{1-\frac{1}{r^{}}erf\left[\frac{1}{m^{}}\left(W_{m}-\sum_{i=1}^{3}C_{i0}{\left(2\lambda^{2}+\lambda^{-4}-3\right)}^{i}\right)\right]\right\}\times & & \\ & & Unload\\ \sum_{i=1}^{3}2iC_{i0}^{}{\left(2\lambda^{2}+\lambda^{-4}-3\right)}^{i-1}(\lambda -\lambda^{-5}) & & \end{array}\right.$$

Similarly, the Ogden pseudo-elastic constitutive model for uniaxial and equal biaxial cases can be obtained by substituting Equations (7), (8) and (12) into Equation (13).

In the case of *ST*, we can obtain:

(16) $$\sigma_{e}^{ST}=\left\{\begin{array}{lll} \sum_{i=1}^{3}\frac{2\mu_{i}^{}}{\alpha_{i}^{}}\left(\lambda^{\alpha_{i}^{}-1}-\lambda^{-\frac{1}{2}\alpha_{i}^{}-1}\right) & & Load\\ \left\{1-\frac{1}{r^{}}erf\left[\frac{1}{m^{}}\left(W_{m}-\sum_{i=1}^{3}\frac{2\mu_{i}^{}}{{\left(\alpha_{i}^{}\right)}^{2}}\left(\lambda^{\alpha_{i}^{}}+2\lambda^{-\frac{1}{2}\alpha_{i}^{}}-3\right)\right)\right]\right\}\times & & \\ & & Unload\\ \sum_{i=1}^{3}\frac{2\mu_{i}^{}}{\alpha_{i}^{}}\left(\lambda^{\alpha_{i}^{}-1}-\lambda^{-\frac{1}{2}\alpha_{i}^{}-1}\right) & & \end{array}\right.$$

In the case of *ET*, we can obtain:

(17) $$\sigma_{e}^{ET}=\left\{\begin{array}{lll} \sum_{i=1}^{3}\frac{2\mu_{i}^{}}{\alpha_{i}^{}}\left(\lambda^{\alpha_{i}^{}-1}-\lambda^{-2\alpha_{i}^{}-1}\right) & & Load\\ \left\{1-\frac{1}{r^{}}erf\left[\frac{1}{m^{}}\left(W_{m}-\sum_{i=1}^{3}\frac{2\mu_{i}^{}}{{\left(\alpha_{i}^{}\right)}^{2}}\left(2\lambda^{\alpha_{i}^{}}+\lambda^{-2\alpha_{i}^{}}-3\right)\right)\right]\right\}\times & & \\ & & Unload\\ \sum_{i=1}^{3}\frac{2\mu_{i}^{}}{\alpha_{i}^{}}\left(\lambda^{\alpha_{i}^{}-1}-\lambda^{-2\alpha_{i}^{}-1}\right) & & \end{array}\right.$$

### 2.3. Constitutive Model of Viscoelastic Unit

In this work, in order to discuss the nonlinear viscoelasticity of materials, it is often impracticable to describe the configuration of the object in the sense of small deformations. Therefore, a general geometric description is required [38]. It can be assumed that in the natural state (zero stress and zero strain state), the object configuration is $X\equiv X\left(X_{1,}X_{2,}X_{3}\right)$ and the reference configuration at the moment t is $x\equiv x\left(x_{1},x_{2},x_{3}\right)$, $x_{i}=x_{i}\left(X,t\right)$,  i=1,2,3. For the sake of simplicity, take $X_{i}$ and $x_{i}$ as the coaxial coordinate system. Then, it can be expressed:s

(18) $$\mathrm{dx}=\frac{\partial x}{\partial X}\mathrm{dX}=F\mathrm{dX}$$

where $F=\left|F_{i,j}\right|$ is called the deformation gradient tensor and its component is $F_{i,j}\equiv \frac{\partial x_{i}}{\partial X_{i}}\equiv x_{i,j}$.

The relative deformation gradient is:

(19) $$F_{t}\left(\tau \right)=\left[\frac{\partial x_{i}\left(\tau \right)}{\partial x_{j}\left(t\right)}\right]$$

The right Cauchy–Green deformation tensor $\;C=\left|C_{KL}\right|$, $C_{KL}\equiv x_{i,K}x_{i,L}$ or

(20) $$C=F^{T}F$$

The right Cauchy–Green relative deformation tensor is:

(21) $$C_{t}(\tau )=F_{t}^{T}F_{t}$$

The left Cauchy–Green deformation tensor or Finger deformation tensor is:

(22) $$B=FF^{T}$$

For incompressible materials such as rubber, the material coordinates and the spatial coordinate system can be set to overlap. The uniform deformation of the general object is expressed as:

(23) $$x_{1}=\lambda_{1}X_{1},\;x_{2}=\lambda_{2}X_{2},\;x_{3}=\lambda_{3}X_{3}$$

where $\lambda_{i}$ is determined by the load and material properties and is independent of the coordinates. The following are special cases of uniaxial and equal biaxial tensile finite deformation of incompressible isotropic materials.

Substituting Equation (2) into Equation (23), the uniaxial and equal biaxial tensile deformation of incompressible isotropic materials can be derived as:

(24) $$\left\{\begin{array}{l} ST:\;x_{1}=\lambda X_{1},x_{2}=\lambda^{-1/2}X_{2},x_{3}=\lambda^{-1/2}X_{3}\\ ET:x_{1}=\lambda X_{1},x_{2}=\lambda X_{2},x_{3}=\lambda^{-2}X_{3} \end{array}\right.$$

where $\lambda =\lambda \left(t\right)$ is the stretch ratio. It is defined as:

(25) $$\lambda =\frac{dx_{1}}{dX_{1}}=1+\frac{dx_{1}-dX_{1}}{dX_{1}}=1+\varepsilon$$

The relevant variables of uniaxial stretching and equal biaxial stretching can be obtained by calculating the coupling based on Equations (18)–(25). The results are as follows.

Regarding the *ST* deformation, we can obtain:

(26) $$F^{ST}=\left[\begin{array}{ccc} \lambda & 0 & 0\\ 0 & \lambda^{-1/2} & 0\\ 0 & 0 & \lambda^{-1/2} \end{array}\right],\;C^{ST}=B^{ST}=\left[\begin{array}{ccc} \lambda^{2} & 0 & 0\\ 0 & \lambda^{-1} & 0\\ 0 & 0 & \lambda^{-1} \end{array}\right]$$

(27) $$F_{t}^{ST}=\left[\begin{array}{ccc} \frac{\lambda \left(\zeta \right)}{\lambda } & 0 & 0\\ 0 & \frac{\lambda^{1/2}}{\lambda^{1/2}\left(\zeta \right)} & 0\\ 0 & 0 & \frac{\lambda^{1/2}}{\lambda^{1/2}\left(\zeta \right)} \end{array}\right],\;\begin{array}{c} C_{t}^{ST}=\left[\begin{array}{ccc} \frac{\lambda^{2}\left(\zeta \right)}{\lambda^{2}} & 0 & 0\\ 0 & \frac{\lambda }{\lambda \left(\zeta \right)} & 0\\ 0 & 0 & \frac{\lambda }{\lambda \left(\zeta \right)} \end{array}\right] \end{array}$$

Regarding the *ET* deformation, we can obtain:

(28) $$F^{ET}=\left[\begin{array}{ccc} \lambda & 0 & 0\\ 0 & \lambda & 0\\ 0 & 0 & \lambda^{-2} \end{array}\right],\;C^{ET}=B^{ET}=\left[\begin{array}{ccc} \lambda^{2} & 0 & 0\\ 0 & \lambda^{2} & 0\\ 0 & 0 & \lambda^{-4} \end{array}\right]$$

(29) $$F_{t}^{ET}=\left[\begin{array}{ccc} \frac{\lambda \left(\zeta \right)}{\lambda } & 0 & 0\\ 0 & \frac{\lambda \left(\zeta \right)}{\lambda } & 0\\ 0 & 0 & \frac{\lambda^{2}}{\lambda^{2}\left(\zeta \right)} \end{array}\right],\;\begin{array}{c} C_{t}^{ET}=\left[\begin{array}{ccc} \frac{\lambda^{2}\left(\zeta \right)}{\lambda^{2}} & 0 & 0\\ 0 & \frac{\lambda^{2}\left(\zeta \right)}{\lambda^{2}} & 0\\ 0 & 0 & \frac{\lambda^{4}}{\lambda^{4}\left(\zeta \right)} \end{array}\right] \end{array}$$

For the constitutive model describing a nonlinear visco-elastic unit, the generalized linear integral theory is adopted in this work. The generalized linear integral theory was developed by Chang, Bloch and Tschoegl [25]. In this theory, an extension of the Seth strain metric is used to link the material properties with the deformation parameters, and a generalized strain function related to material properties is introduced, greatly simplifying the constitutive equation. Within this theoretical framework, the relationship between stress and deformation for incompressible solid polymers can be expressed as:

(30) $$\sigma \left(t\right)=-pI+\frac{1}{\tilde{n}}\int_{-\infty }^{t}G\left(t-\zeta \right)\left[B^{\frac{\tilde{n}}{2}}\left(t\right)\frac{d}{d\zeta }C_{t}^{\frac{\tilde{n}}{2}}\left(\zeta \right)+\frac{d}{d\zeta }C_{t}^{\frac{\tilde{n}}{2}}\left(\zeta \right)B^{\frac{\tilde{n}}{2}}\left(t\right)\right]d\zeta$$

where $G\left(t\right)$ is the relaxation modulus; I is the unit matrix; and a symmetric tensor is given in square brackets. $\tilde{n}$ is the strain parameter and material parameter, depending on many factors including materials and temperature, and it is generally not an integer. When $\tilde{n}=2$, Equation (30) can be simplified as [25]:

(31) $$\sigma \left(t\right)=-pI+\frac{1}{2}\int_{-\infty }^{t}G\left(t-\zeta \right)\left[B\left(t\right)\frac{d}{d\zeta }C_{t}\left(\zeta \right)+\frac{d}{d\zeta }C_{t}^{}\left(\zeta \right)B\left(t\right)\right]d\zeta$$

For uniaxial stretching, substituting Equations (26) and (27) into Equation (31) and using the condition of $\sigma_{22}=\sigma_{33}=0$, we obtain:

(32) $$\sigma_{11}^{ST}\left(t\right)=\int_{-\infty }^{t}G(t-\zeta )\frac{d}{d\zeta }\left\{\lambda^{2}(\zeta )-\frac{1}{\lambda \left(\zeta \right)}\right\}d\zeta$$

For equal biaxial stretching, substituting Equations (28) and (29) into Equation (31) and using the condition of $\sigma_{33}=0$, we obtain:

(33) $$\sigma_{11}^{ET}\left(t\right)=\sigma_{22}^{ET}\left(t\right)=\int_{-\infty }^{t}G\left(t-\zeta \right)\frac{d}{d\zeta }\left\{\lambda^{2}\left(\zeta \right)-{\left[\frac{1}{\lambda \left(\zeta \right)}\right]}^{4}\right\}d\zeta$$

where $G\left(t\right)$ is the relaxation modulus, which can be described by the Prony series to facilitate simulation, and its expression is as follows [39]:

(34) $$G\left(t\right)=E_{\infty }+\sum_{i=1}^{n}E_{i}e^{-t/\tau_{i}}\;\begin{array}{c} \left(n=1,2,3,\ldots \right) \end{array}$$

where, $E_{i}$ is the modulus, $\tau_{i}$ is the relaxation time parameter, and $E_{\infty }$ is the equilibrium modulus, which is the steady-state response of the relaxation modulus at t→∞. In this work, a Prony series of the second order $\left(n=2\right)$ is chosen for the viscoelastic unit model.

When a constant strain is loaded suddenly, $\lambda \left(t\right)$ can be expressed as:

(35) $$\lambda \left(t\right)=\left\{\begin{array}{l} 1\begin{array}{cc} & t\leq 0 \end{array}\\ \lambda_{0}\begin{array}{cc} & t>0 \end{array} \end{array}\right.$$

where $\lambda_{0}$ is the sudden constant stretch ratio.

Substituting Equations (34) and (35) into Equations (32) and (33), the stress relaxation function can be derived as follows:

(36) $$\left\{\begin{array}{l} ST:\sigma_{t}^{ST}\left(t\right)=\left(\lambda_{0}^{2}-\lambda_{0}^{-1}\right)\times \left(E_{\infty }+\sum_{i=1}^{2}E_{i}e^{-t/\tau_{i}}\right)\\ ET:\sigma_{t}^{ET}\left(t\right)=\left(\lambda_{0}^{2}-\lambda_{0}^{-4}\right)\times \left(E_{\infty }+\sum_{i=1}^{2}E_{i}e^{-t/\tau_{i}}\right) \end{array}\right.$$

Then, for the constitutive model of the viscoelastic unit at loading, the integration of Equation (25) into Equations (32) and (33) yields:

(37) $$\left\{\begin{array}{l} ST:\sigma_{V}^{ST}\left(t\right)=2{\dot{\varepsilon }}_{L}^{}\int_{0}^{t}G\left(t-\zeta \right)\lambda \left(\zeta \right)d\zeta +{\dot{\varepsilon }}_{L}^{}\int_{0}^{t}G\left(t-\zeta \right)\lambda^{-2}\left(\zeta \right)d\zeta \\ ET:\sigma_{V}^{ET}\left(t\right)=2{\dot{\varepsilon }}_{L}^{}\int_{0}^{t}G\left(t-\zeta \right)\lambda \left(\zeta \right)d\zeta +4{\dot{\varepsilon }}_{L}^{}\int_{0}^{t}G\left(t-\zeta \right)\lambda^{-5}\left(\zeta \right)d\zeta \end{array}\right.$$

where ${\dot{\varepsilon }}_{L}$ is the strain rate at loading, which is a constant; and $\sigma_{v}$ is the time-dependent nonlinear visco-elastic stress.

To perform an integral operation on Equation (37), the following Equation (38) is added:

(38) $$\left\{\begin{array}{l} Q_{1}\left(t\right)=\int_{0}^{t}G\left(t-\zeta \right)\lambda \left(\zeta \right)d\zeta \\ Q_{2}\left(t\right)=\int_{0}^{t}G\left(t-\zeta \right)\lambda^{-2}\left(\zeta \right)d\zeta \\ Q_{3}\left(t\right)=\int_{0}^{t}G\left(t-\zeta \right)\lambda^{-5}\left(\zeta \right)d\zeta \end{array}\right.$$

Substituting Equation (34) into Equation (38) and performing integration, we can obtain:

(39) $$\left\{\begin{array}{l} Q_{1}\left(t\right)=E_{\infty }t\left(\lambda -\frac{1}{2}{\dot{\varepsilon }}_{L}t\right)+\sum_{i=1}^{2}E_{i}\left[\tau_{i}\left(\lambda -e^{-t/\tau_{i}}\right)-{\dot{\varepsilon }}_{L}\tau_{i}^{2}\left(1-e^{-t/\tau_{i}}\right)\right]\\ Q_{2}\left(t\right)=\frac{E_{\infty }}{{\dot{\varepsilon }}_{L}}\left(1-\frac{1}{\lambda }\right)+\sum_{i=1}^{2}\frac{E_{i}t}{\sum_{k=1}^{3}\left[\frac{1}{3}\prod_{m=1}^{k}\left(4-m\right)\right]{\dot{\varepsilon }}_{L}{}^{k-1}\tau_{i}^{k}\left(e^{t/\tau_{i}}-\lambda^{3-k}\right)}\\ Q_{3}\left(t\right)=\frac{E_{\infty }}{4{\dot{\varepsilon }}_{L}}\left(1-\frac{1}{\lambda^{4}}\right)+\sum_{i=1}^{2}\frac{E_{i}t}{\sum_{k=1}^{6}\left[\frac{1}{6}\prod_{m=1}^{k}\left(7-m\right)\right]{\dot{\varepsilon }}_{L}{}^{k-1}\tau_{i}^{k}\left(e^{t/\tau_{i}}-\lambda^{6-k}\right)} \end{array}\right.$$

Substituting Equations (38) and (39) into Equation (37), the constitutive model of the viscoelastic unit for uniaxial tensile and equal biaxial tensile at loading can be obtained:

(40) $$\left\{\begin{array}{l} ST:\sigma_{V}^{ST}\left(t\right)=2{\dot{\varepsilon }}_{L}^{}Q_{1}\left(t\right)+{\dot{\varepsilon }}_{L}^{}Q_{2}\left(t\right)\\ ET:\sigma_{V}^{ET}\left(t\right)=2{\dot{\varepsilon }}_{L}^{}Q_{1}\left(t\right)+4{\dot{\varepsilon }}_{L}^{}Q_{3}\left(t\right) \end{array}\right.$$

We let $0-t_{1}$ be the loading time and $t_{1}-t$ be the unloading time. For the constitutive model of viscoelastic unit at unloading, it is necessary to consider the entire loading and unloading history, as shown in Equation (41).

(41) $$\left\{\begin{array}{l} ST:\sigma_{V}^{ST}\left(t\right)=\int_{0}^{t_{1}}G(t_{1}-\zeta )\frac{d}{d\zeta }\left\{\lambda^{2}(\zeta )-\frac{1}{\lambda \left(\zeta \right)}\right\}d\zeta +\\ \int_{t_{1}}^{t}G(t-\zeta )\frac{d}{d\zeta }\left\{\lambda^{2}(\zeta )-\frac{1}{\lambda \left(\zeta \right)}\right\}d\zeta \\ ET:\sigma_{V}^{ET}\left(t\right)=\int_{0}^{t_{1}}G\left(t_{1}-\zeta \right)\frac{d}{d\zeta }\left\{\lambda^{2}\left(\zeta \right)-{\left[\frac{1}{\lambda \left(\zeta \right)}\right]}^{4}\right\}d\zeta +\\ \int_{t_{1}}^{t}G\left(t-\zeta \right)\frac{d}{d\zeta }\left\{\lambda^{2}\left(\zeta \right)-{\left[\frac{1}{\lambda \left(\zeta \right)}\right]}^{4}\right\}d\zeta \end{array}\right.$$

Substituting Equation (25) into Equation (41) and performing integration. The constitutive model for viscoelastic at unloading yields:

(42) $$\left\{\begin{array}{l} ST:\sigma_{V}^{ST}\left(t\right)=2{\dot{\varepsilon }}_{L}^{ST}\int_{0}^{t_{1}}G\left(t_{1}-\zeta \right)\lambda \left(\zeta \right)d\zeta +{\dot{\varepsilon }}_{L}^{ST}\int_{0}^{t_{1}}G\left(t_{1}-\zeta \right)\lambda^{-2}\left(\zeta \right)d\zeta +\\ 2{\dot{\varepsilon }}_{U}^{ST}\int_{t_{1}}^{t}G\left(t-\zeta \right)\lambda \left(\zeta \right)d\zeta +{\dot{\varepsilon }}_{U}^{ST}\int_{t_{1}}^{t}G\left(t-\zeta \right)\lambda^{-2}\left(\zeta \right)d\zeta \\ ET:\sigma_{V}^{ET}\left(t\right)=2{\dot{\varepsilon }}_{L}^{ET}\int_{0}^{t_{1}}G\left(t_{1}-\zeta \right)\lambda \left(\zeta \right)d\zeta +4{\dot{\varepsilon }}_{L}^{ET}\int_{0}^{t_{1}}G\left(t_{1}-\zeta \right)\lambda^{-5}\left(\zeta \right)d\zeta +\\ 2{\dot{\varepsilon }}_{U}^{ET}\int_{t_{1}}^{t}G\left(t-\zeta \right)\lambda \left(\zeta \right)d\zeta +4{\dot{\varepsilon }}_{U}^{ET}\int_{t_{1}}^{t}G\left(t-\zeta \right)\lambda^{-5}\left(\zeta \right)d\zeta \end{array}\right.$$

where ${\dot{\varepsilon }}_{U}$ is the strain rate at unloading, which is also a constant; and $t_{1}$ is the maximum time of loading.

To perform an integral operation on Equation (42), the following Equation (43) is added:

(43) $$\left\{\begin{array}{l} Q_{4}\left(t\right)=\int_{t_{1}}^{t}G\left(t-\zeta \right)\lambda \left(\zeta \right)d\zeta \\ Q_{5}\left(t\right)=\int_{t_{1}}^{t}G\left(t-\zeta \right)\lambda^{-2}\left(\zeta \right)d\zeta \\ Q_{6}\left(t\right)=\int_{t_{1}}^{t}G\left(t-\zeta \right)\lambda^{-5}\left(\zeta \right)d\zeta \end{array}\right.$$

Substituting Equation (34) into Equation (43) and performing integration, we can obtain:

(44) $$\left\{\begin{array}{l} Q_{4}\left(t\right)=E_{\infty }\left[\left(\lambda t-\lambda_{1}t_{1}\right)-\frac{1}{2}{\dot{\varepsilon }}_{U}\left(t^{2}-t_{1}{}^{2}\right)\right]+\sum_{i=1}^{2}E_{i}\left[\tau_{i}\left(\lambda -\lambda_{1}e^{-\left(t-t_{1}\right)/\tau_{i}}\right)-\right.\\ \begin{array}{ccc} & & \tau_{i}^{2}{\dot{\varepsilon }}_{U} \end{array}\left.\left(1-e^{-\left(t-t_{1}\right)/\tau_{i}}\right)\right]\\ Q_{5}\left(t\right)=\frac{E_{\infty }}{{\dot{\varepsilon }}_{U}}\left(\frac{1}{\lambda_{1}}-\frac{1}{\lambda }\right)+\sum_{i=1}^{2}\frac{E_{i}\left(t-t_{1}\right)}{\sum_{k=1}^{3}\left[\frac{1}{3}\prod_{m=1}^{k}\left(4-m\right)\right]{\dot{\varepsilon }}_{U}{}^{k-1}\tau_{i}^{k}\left(e^{\left(t-t_{1}\right)/\tau_{i}}\lambda_{1}^{3-k}-\lambda^{3-k}\right)}\\ Q_{6}\left(t\right)=\frac{E_{\infty }}{4{\dot{\varepsilon }}_{U}}\left(\frac{1}{\lambda^{4}{}_{1}}-\frac{1}{\lambda^{4}}\right)+\sum_{i=1}^{2}\frac{E_{i}\left(t-t_{1}\right)}{\sum_{k=1}^{6}\left[\frac{1}{6}\prod_{m=1}^{k}\left(7-m\right)\right]{\dot{\varepsilon }}_{U}{}^{k-1}\tau_{i}^{k}\left(e^{\left(t-t_{1}\right)/\tau_{i}}\lambda_{1}^{6-k}-\lambda^{6-k}\right)} \end{array}\right.$$

where $\lambda_{1}$ is the maximum stretch ratio at loading.

Substituting Equations (43) and (44) into Equation (42), the constitutive model of the viscoelastic unit for uniaxial tension and equal biaxial tension unloading can be obtained:

(45) $$\left\{\begin{array}{l} ST:\sigma_{V}^{ST}\left(t\right)=2{\dot{\varepsilon }}_{L}Q_{1}\left(t_{1}\right)+{\dot{\varepsilon }}_{L}Q_{2}\left(t_{1}\right)+2{\dot{\varepsilon }}_{U}Q_{4}\left(t\right)+{\dot{\varepsilon }}_{U}Q_{5}\left(t\right)\\ ET:\sigma_{V}^{ET}\left(t\right)=2{\dot{\varepsilon }}_{L}Q_{1}\left(t_{1}\right)+4{\dot{\varepsilon }}_{L}Q_{3}\left(t_{1}\right)+2{\dot{\varepsilon }}_{U}Q_{4}\left(t\right)+4{\dot{\varepsilon }}_{U}Q_{6}\left(t\right) \end{array}\right.$$

### 2.4. Construction of the Visco-Hyperelastic Constitutive Model

#### 2.4.1. The Visco-Hyperelastic Constitutive Model of Yeoh Type

The constitutive model of the hyper-elastic unit and the constitutive model of the visco-elastic unit can be obtained by combining Equations (14), (15), (40) and (45). Then, according to Equation (1), we construct a visco-hyperelastic constitutive model of Yeoh type for the uniaxial and equal biaxial cases, as shown in Equations (46) and (47).

In the case of *ST*, we can obtain:

(46) $$\sigma^{ST}=\left\{\begin{array}{lll} \sum_{i=1}^{3}2iC_{i0}^{}{\left(\lambda^{2}+2\lambda^{-1}-3\right)}^{i-1}(\lambda -\lambda^{-2})+2{\dot{\varepsilon }}_{L}Q_{1}\left(t\right)+{\dot{\varepsilon }}_{L}Q_{2}\left(t\right) & & Load\\ \left\{1-\frac{1}{r^{}}erf\left[\frac{1}{m}\left(W_{m}-\sum_{i=1}^{3}C_{i0}{\left(\lambda^{2}+2\lambda^{-1}-3\right)}^{i}\right)\right]\right\}\times & & \\ \sum_{i=1}^{3}2iC_{i0}^{}{\left(\lambda^{2}+2\lambda^{-1}-3\right)}^{i-1}(\lambda -\lambda^{-2})+2{\dot{\varepsilon }}_{L}Q_{1}\left(t_{1}\right)+{\dot{\varepsilon }}_{L}Q_{2}\left(t_{1}\right)+ & & Unload\\ 2{\dot{\varepsilon }}_{U}Q_{4}\left(t\right)+{\dot{\varepsilon }}_{U}Q_{5}\left(t\right) & & \end{array}\right.$$

In the case of *ET*, we can obtain:

(47) $$\sigma^{ET}=\left\{\begin{array}{lll} \sum_{i=1}^{3}2iC_{i0}^{}{\left(2\lambda^{2}+\lambda^{-4}-3\right)}^{i-1}(\lambda -\lambda^{-5})+2{\dot{\varepsilon }}_{L}Q_{1}\left(t\right)+4{\dot{\varepsilon }}_{L}Q_{3}\left(t\right) & & Load\\ \left\{1-\frac{1}{r^{}}erf\left[\frac{1}{m^{}}\left(W_{m}-\sum_{i=1}^{3}C_{i0}{\left(2\lambda^{2}+\lambda^{-4}-3\right)}^{i}\right)\right]\right\}\times & & \\ \sum_{i=1}^{3}2iC_{i0}^{}{\left(2\lambda^{2}+\lambda^{-4}-3\right)}^{i-1}(\lambda -\lambda^{-5})+2{\dot{\varepsilon }}_{L}Q_{1}\left(t_{1}\right)+4{\dot{\varepsilon }}_{L}Q_{3}\left(t_{1}\right)+ & & Unload\\ 2{\dot{\varepsilon }}_{U}Q_{4}\left(t\right)+4{\dot{\varepsilon }}_{U}Q_{6}\left(t\right) & & \end{array}\right.$$

#### 2.4.2. The Visco-Hyperelastic Constitutive Model of Ogden Type

Likewise, combining Equations (16), (17), (40) and (45), and then, according to Equation (1), the visco-hyperelastic constitutive model of Ogden type can be constructed, as shown in Equations (48) and (49).

In the case of *ST*, we can obtain:

(48) $$\sigma^{ST}=\left\{\begin{array}{lll} \sum_{i=1}^{3}\frac{2\mu_{i}^{}}{\alpha_{i}^{}}\left(\lambda^{\alpha_{i}^{}-1}-\lambda^{-\frac{1}{2}\alpha_{i}^{}-1}\right)+2{\dot{\varepsilon }}_{L}Q_{1}\left(t\right)+{\dot{\varepsilon }}_{L}Q_{2}\left(t\right) & & Load\\ \left\{1-\frac{1}{r^{}}erf\left[\frac{1}{m^{}}\left(W_{m}-\sum_{i=1}^{3}\frac{2\mu_{i}^{}}{{\left(\alpha_{i}^{}\right)}^{2}}\left(\lambda^{\alpha_{i}^{}}+2\lambda^{-\frac{1}{2}\alpha_{i}^{}}-3\right)\right)\right]\right\}\times & & \\ \sum_{i=1}^{3}\frac{2\mu_{i}^{}}{\alpha_{i}^{}}\left(\lambda^{\alpha_{i}^{}-1}-\lambda^{-\frac{1}{2}\alpha_{i}^{}-1}\right)+2{\dot{\varepsilon }}_{L}Q_{1}\left(t_{1}\right)+{\dot{\varepsilon }}_{L}Q_{2}\left(t_{1}\right)+ & & Unload\\ 2{\dot{\varepsilon }}_{U}Q_{4}\left(t\right)+{\dot{\varepsilon }}_{U}Q_{5}\left(t\right) & & \end{array}\right.$$

In the case of *ET*, we can obtain:

(49) $$\sigma^{ET}=\left\{\begin{array}{lll} \sum_{i=1}^{3}\frac{2\mu_{i}^{}}{\alpha_{i}^{}}\left(\lambda^{\alpha_{i}^{}-1}-\lambda^{-2\alpha_{i}^{}-1}\right)+2{\dot{\varepsilon }}_{L}Q_{1}\left(t\right)+4{\dot{\varepsilon }}_{L}Q_{3}\left(t\right) & & Load\\ \left\{1-\frac{1}{r}erf\left[\frac{1}{m}\left(W_{m}-\sum_{i=1}^{3}\frac{2\mu_{i}}{{\left(\alpha_{i}^{}\right)}^{2}}\left(2\lambda^{\alpha_{i}^{}}+\lambda^{-2\alpha_{i}^{}}-3\right)\right)\right]\right\}\times & & \\ \sum_{i=1}^{3}\frac{2\mu_{i}^{}}{\alpha_{i}^{}}\left(\lambda^{\alpha_{i}^{}-1}-\lambda^{-2\alpha_{i}^{}-1}\right)+2{\dot{\varepsilon }}_{L}Q_{1}\left(t_{1}\right)+4{\dot{\varepsilon }}_{L}Q_{3}\left(t_{1}\right)+ & & Unload\\ 2{\dot{\varepsilon }}_{U}Q_{4}\left(t\right)+4{\dot{\varepsilon }}_{U}Q_{6}\left(t\right) & & \end{array}\right.$$

## 3. Experimental Procedure

### 3.1. Test Material

The test material was an EPDM rubber sheet, with a Shore’s hardness of around 50–55 degrees from Nanjing Dongrun Special Rubber and Plastic Co., Ltd. (Nanjing, China), which meets the testing standards product qualification certificate of GB/T5574-2008 [40]. The test material was prepared mainly through the following steps: firstly, the rubber was refined, then mechanically extruded, and finally air-cooled at room temperature. The composition of the EPDM rubber in this study is shown in Table 1.

**Figure 2:**
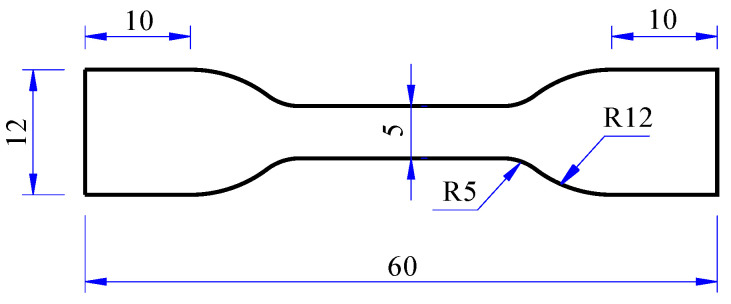
Uniaxial tensile dumbbell specimen (units: mm).

**Figure 3:**
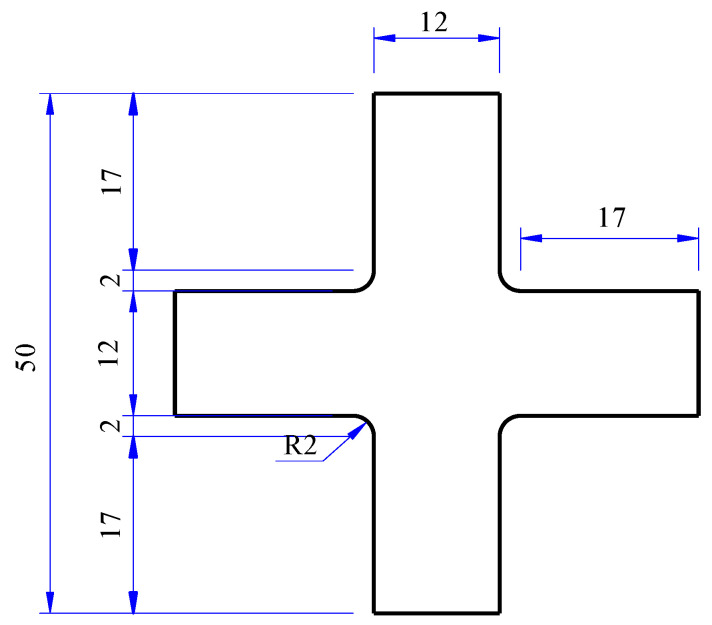
Biaxial tensile cross specimen (units: mm).

The specimens of uniaxial tensile dumbbell type and biaxial tensile cross type were obtained by using specialized dumbbell-shaped and cross-shaped cutters to ensure dimensional accuracy and integrity of the specimen. The shape and size of the specimens are shown in Figure 2 and Figure 3. The thickness of the specimens was 2 mm.

### 3.2. Test Schemes

The testing instrument adopted was the Care IPBF-300 in situ biaxial fatigue testing system produced by Care Measurement and Control (Tianjin) Co., Ltd. (Tianjin, China). A non-contact video extensometer and light source were included in the system to enable real-time measurement of the bi-directional deformation in the specimen’s central region. In addition to realizing uniaxial independent testing, the system can realize biaxial equal proportional, non-proportional loading, biaxial synchronous tensile, cyclic, asynchronous loading, and so on.

**Figure 4:**
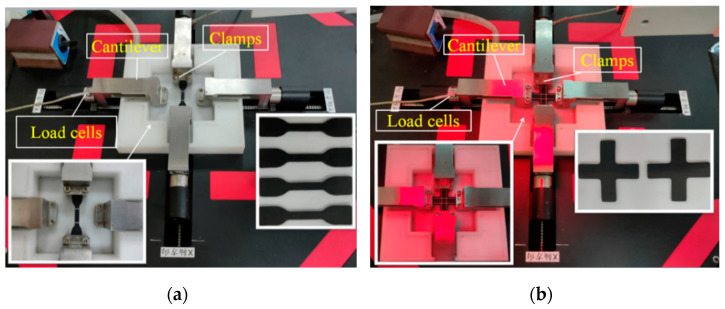
Care IPBF-300 in situ biaxial fatigue test system: (**a**) used for uniaxial tensile tests of EPDM rubber; (**b**) used for biaxial tensile tests of EPDM rubber.

The testing system with EPDM specimens is shown in Figure 4. This work involves two types of single-stage cycles with different displacements and one type of three-stage cycle. In order to facilitate the distinction, the cycle with smaller tensile deformation is named as A-type cycle, the cycle with larger tensile deformation as B-type cycle, and the multi-stage cycle as C-type cycle. The uniaxial and equal biaxial stress relaxation test scheme is shown in Table 2. The uniaxial and equal biaxial cyclic tensile test scheme is shown in Table 3. The uniaxial and biaxial tensile tests were performed on the EPDM specimen with white marking lines at a room temperature of around 24 °C. During the testing process, the loading equipment simultaneously recorded the load, displacement, and time. The non-contact video extensometer synchronously measured the deformation of the testing zone by recognizing the marking lines of the specimen in real time based on the digital image technology.

### 3.3. Test Results

**Figure 5:**
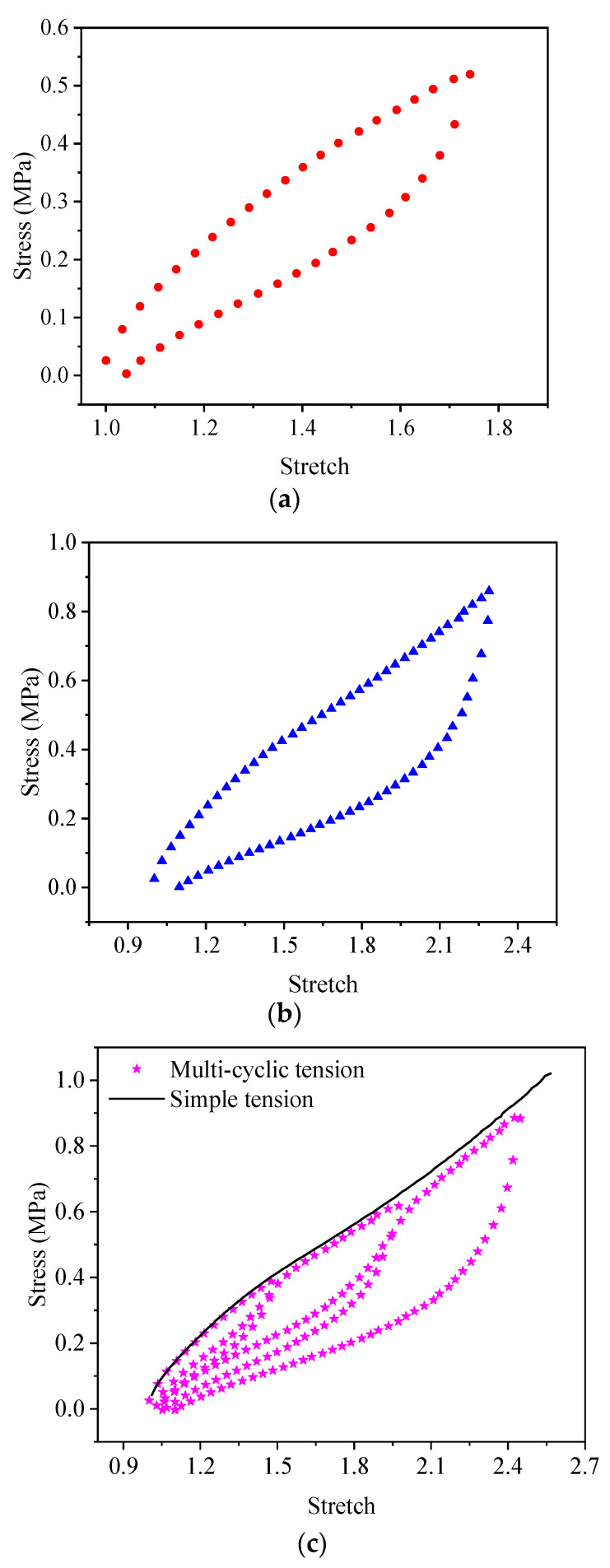
Uniaxial cyclic test results of EPDM rubber: (**a**) A-type cycle; (**b**) B-type cycle; (**c**) C-type cycle and simple tension.

The test curves for the uniaxial cycling tests of the EPDM material are shown in Figure 5. As seen in Figure 5c, when the EPDM material undergoes the load–unload–reload cycle, the unloading stress and reloading stress are much lower than the stress at the first loading. Moreover, as the stretch exceeds the maximum tensile amount previously applied, the stretch curve of the EPDM material continues to follow the simple tensile test curve (uniaxial main loading curve) after a short transition, which indicates that EPDM presents a significant Mullins softening effect under uniaxial tension.

**Figure 6:**
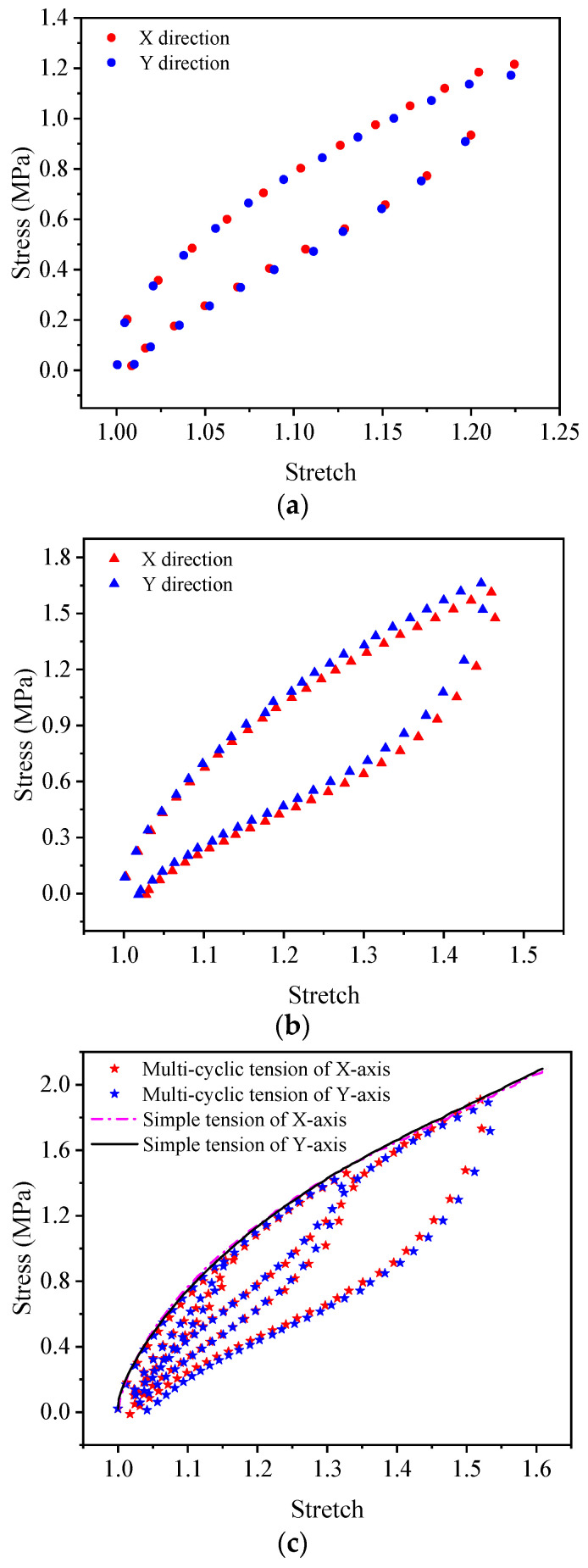
The equal biaxial cycle test results of EPDM rubber: (**a**) A-type cycle; (**b**) B-type cycle; (**c**) C-type cycle and simple tension.

Figure 6 shows the experiment results for EPDM in the equal biaxial cyclic tests. In the equal biaxial tests of EPDM, the test curves in the X-direction and Y-direction are almost coincident, indicating that the EPDM material used in the experiment is isotropic. In addition, according to the test results in Figure 6c, the EPDM material also shows an obvious Mullins effect in the equal biaxial stress state.

It is worth pointing out that the deformation of the EPDM material does not recover simultaneously with the stress unloaded to zero either in the uniaxial tensile tests (Figure 5) or the biaxial tensile tests (Figure 6), which is due to the viscoelasticity of EPDM rubber.

**Figure 7:**
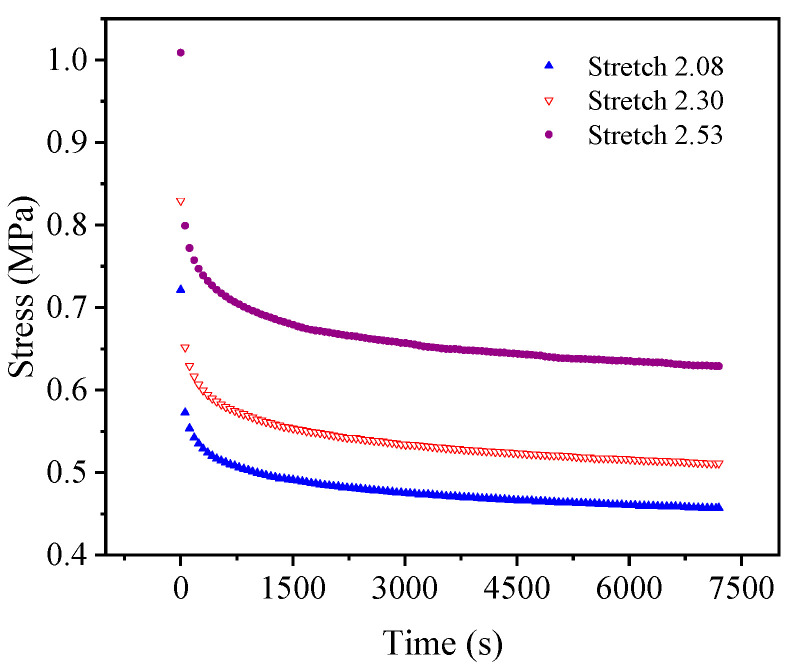
Uniaxial stress relaxation test results of EPDM rubber.

**Figure 8:**
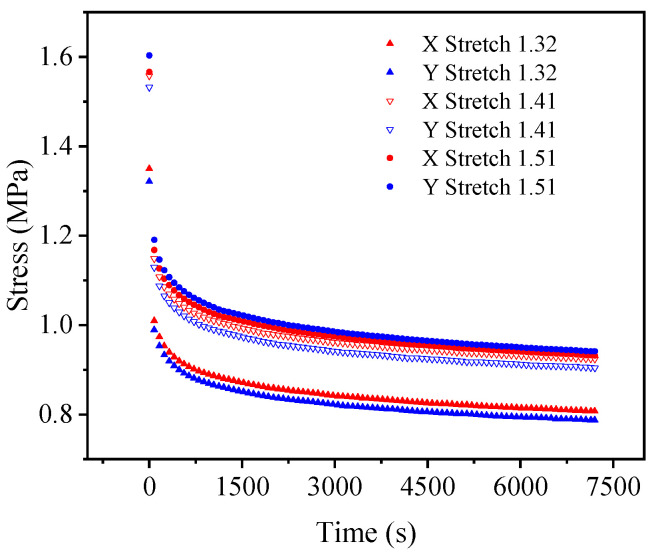
Equal biaxial stress relaxation test results of EPDM rubber.

Figure 7 and Figure 8 show the uniaxial and biaxial stress relaxation test curves, respectively, of the EPDM material under three different constant displacement conditions. It can be seen from the figures that when the stretch ratio of the EPDM material is held, the stress of the material decreases dramatically with time at the beginning of the stress relaxation tests and gradually tends to change slowly under both uniaxial and biaxial stretching. This indicates that the EPDM material has obvious stress relaxation phenomena, whether under uniaxial tension or biaxial tension, revealing its remarkable viscoelasticity. Therefore, in order to accurately describe the mechanical behavior of the EPDM material, its viscoelasticity should be considered.

Moreover, from Figure 8, it can be seen that under the same constant tensile ratio, the stress relaxation test curves in the X and Y directions are approximately coincident, which indicates once more that the EPDM material used in the experiment is isotropic. For simplicity, the following calculation and analysis of the equal biaxial test results take the mean value of X-direction and Y-tensile direction, and the following research mainly focuses on the A-type and B-type cycles.

## 4. Model Fitting and Analysis

### 4.1. Determination of Parameters of the Proposed Visco-Hyperelastic Constitutive Model

In this research, the basic process for determining the parameters of the proposed visco-hyperelastic constitutive model includes two main steps. Step 1: The relaxation function (Equation (36)) is employed to fit the stress relaxation test data to determine the model parameters of the visco-elastic unit. Step 2: Combined with the obtained visco-elastic parameters, the proposed visco-hyperelastic constitutive model (Equations (46)–(49)) is used to approximate the cyclic test data to determine the other parameters (the parameters of the hyper-elastic unit) of the proposed model. In step 2, the loading portion of the cyclic test curve is first fitted to determine the parameters at loading, and then the unloading portion of the cyclic test curve is fitted together with the parameters at loading to determine the parameters at unloading. In order to obtain the optimal parameters of the proposed visco-hyperelastic constitutive model, a goodness of fit was used as a measure to evaluate the degree of congruence of the constitutive model with a coefficient of determination *R*^2^ [41]:

(50) $$R^{2}=1-\frac{SSE}{SST}$$

where *SSE* is the residual sum of squares ($SSE=\sum_{i=1}^{N}{\left({\hat{y}}_{i}-y_{i}\right)}^{2}$); and *SST* is the sum of squared total deviations ($SST=\sum_{i=1}^{N}{\left({\bar{y}}_{i}-y_{i}\right)}^{2}$), where $\hat{y}$ is the fitted value, $y_{i}$ is the test value, and ${\bar{y}}_{i}$ is the mean value. The closer the value of $R^{2}$ is to 1, the better the fit is.

The loading and unloading strain rates for uniaxial and equal biaxial tension can be derived from the above experimental results, as shown in Table 4. The stress relaxation function is employed to approximate the data of the stress relaxation tests. Then, the model parameters of the viscoelastic unit are obtained, as shown in Table 5.

According to step 2 above, combined with the parameters of the viscoelastic unit in Table 5, the parameters of the hyper-elastic unit of the proposed visco-hyperelastic constitutive model of Yeoh type for uniaxial and equal biaxial cases of EPDM rubber can be determined, as shown in Table 6. In the same way, the parameters of the proposed model of Ogden type for uniaxial and equal biaxial cases of EPDM rubber can be also determined, which are presented in Table 7.

According to the energy dissipation loss  D=∮σdε [42], where ε=λ−1, The areas of stress-strain hysteresis loop in Figure 5 and Figure 6 can be calculated, and the energy dissipation losses of A-type cycle and B-type cycle under uniaxial stress state are obtained as 102,052 J/m^3^ and 343,840 J/m^3^. The energy dissipation losses of A-type cycle and B-type cycle under biaxial stress state are obtained as 62,398 J/m^3^ and 227,161 J/m^3^. Linking them with the parameters *r* and *m*, it can be seen from Table 6 and Table 7 that, as the hysteresis loop increases, the energy dissipation of EPDM rubber increases, while the parameters *r* and *m* decrease in both uniaxial and equal biaxial cases.

In addition, from Table 5, Table 6 and Table 7, it can be seen that the coefficient of determination *R*^2^ of the goodness of fit in this study are all greater than 0.97, which suggests the proposed visco-hyperelastic constitutive model is in good agreement with the experimental data.

**Figure 9:**
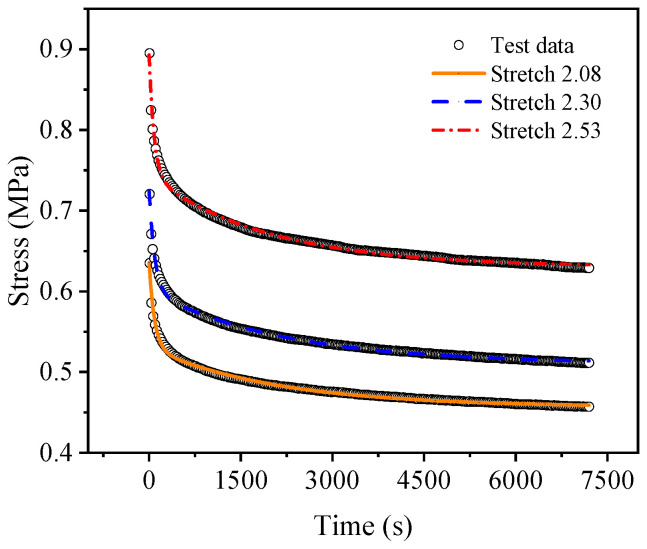
Comparison of fitting results with experimental curves of uniaxial stress relaxation.

**Figure 10:**
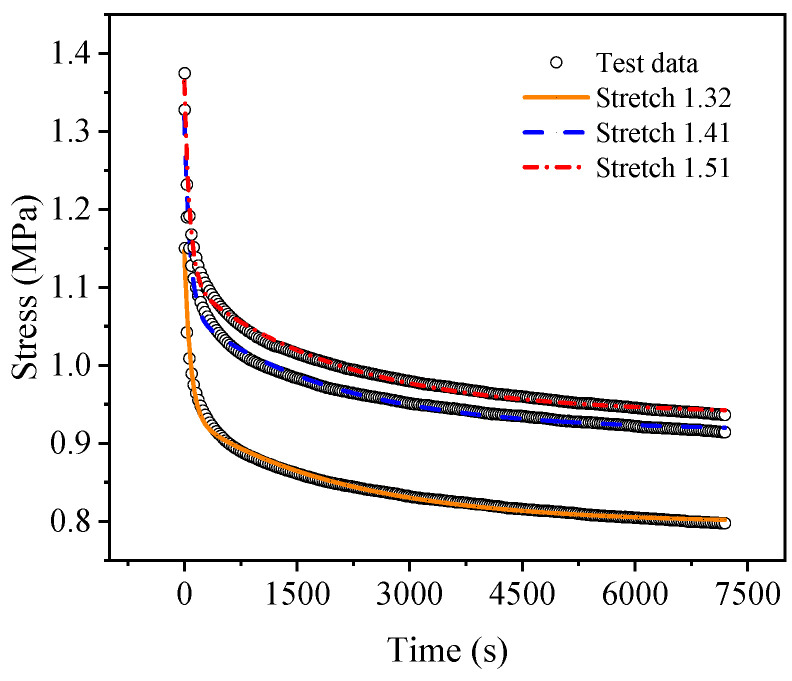
Comparison of fitting results with experimental curves of equi-biaxial stress relaxation.

**Figure 11:**
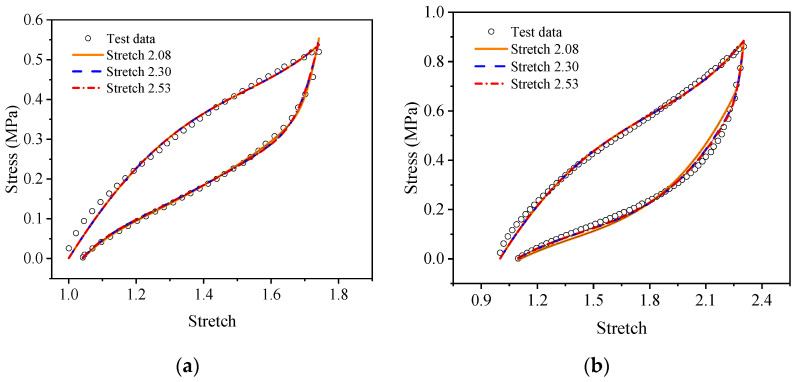
The fitting results of the proposed visco-hyperelastic constitutive model of Yeoh type under uniaxial tension: (**a**) A-type cycle; (**b**) B-type cycle.

**Figure 12:**
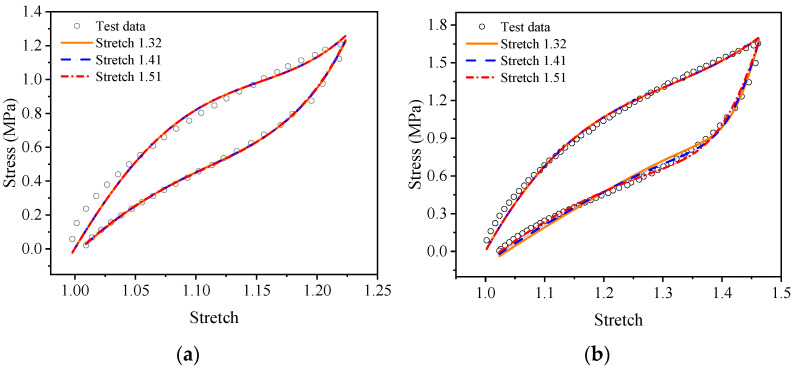
The fitting results of the proposed visco-hyperelastic constitutive model with of type under equi-biaxial tension: (**a**) A-type cycle; (**b**) B-type cycle.

**Figure 13:**
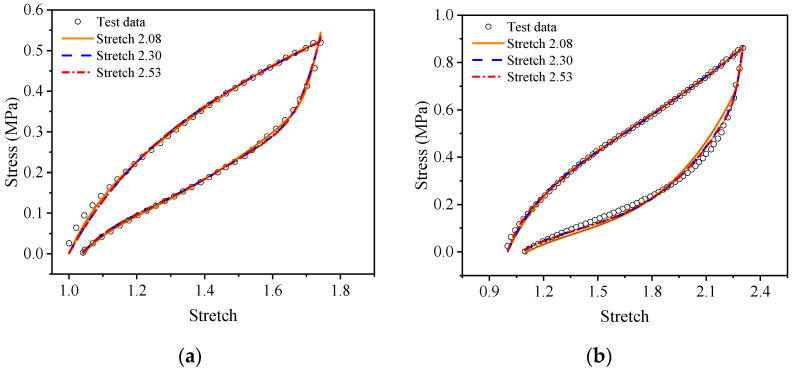
The fitting results of the proposed visco-hyperelastic constitutive model of Ogden type under uniaxial tension: (**a**) A-type cycle; (**b**) B-type cycle.

**Figure 14:**
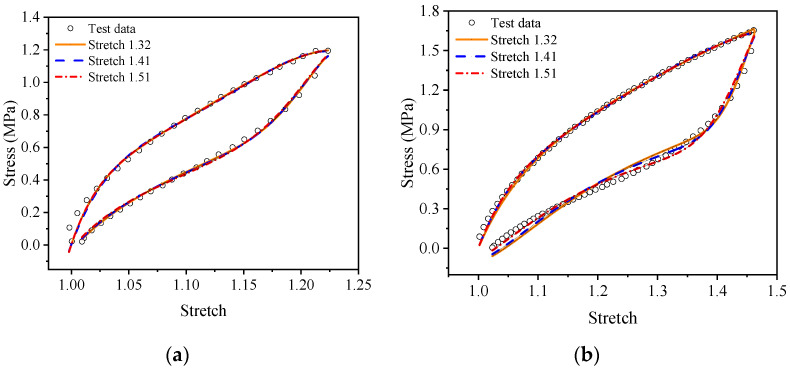
The fitting results of the proposed visco-hyperelastic constitutive model of Ogden type under equi-biaxial tension: (**a**) A-type cycle; (**b**) B-type cycle.

For a more intuitive visual comparison and analysis of models and experimental data, the experimental curves and the fitting results are plotted, as shown in Figure 9, Figure 10, Figure 11, Figure 12, Figure 13 and Figure 14. From Figure 9 and Figure 10, it can be clearly seen that the stress relaxation function in this work fits the test data of EPDM rubber well under three types of stretch ratio levels.

As shown in Figure 11 and Figure 12, it can be seen that the fitting results of the proposed visco-hyperelastic constitutive model of Yeoh type are in good agreement with the cyclic stretching test data of EPDM rubber under uniaxial and equi-biaxial conditions, regardless of whether any constant stretch ratio is used.

Meanwhile, from Figure 13 and Figure 14, it can be seen that the proposed visco-hyperelastic constitutive model of Ogden type also presents a good approximation to both A-type cyclic stretching tests and B-type cyclic stretching tests of EPDM rubber under uniaxial and equal biaxial conditions, regardless of whether one of the constant stretch ratios is used. Moreover, combined with Table 7, it can be seen that for A-type cyclic uniaxial stretching tests, when the stress relaxation test data with a stretch ratio of 2.08 are applied, the fitting effect is the best. The corresponding coefficients of determination, *R*^2^, of the loading and unloading stages are 0.9966 and 0.9978, respectively, which are close to 1. For the A-type cyclic equal biaxial stretching tests, the corresponding optimal constant stretch ratio is 1.32. Similarly, for the B-type cyclic stretching tests under uniaxial and equal biaxial conditions, the corresponding optimal constant stretch ratios are 2.53 and 1.51, respectively. According to Figure 13 and Figure 14, the maximum stretch ratios of A-type cyclic tests under uniaxial and equal biaxial condition are 1.76 and 1.23, respectively. The maximum stretch ratios of B-type cyclic tests are 2.42 and 1.47, respectively. Based on these results, we can conclude that the proposed model has the best fitting effect when the constant stretch ratio of the selected stress relaxation test is closest to the maximum stretch ratio of the cyclic test. In fact, the coefficients of determination of goodness of fit are all around 0.98 (Table 7). Generally, as long as the constant tensile ratio of the selected stress relaxation test is close to the maximum tensile ratio of the cyclic test, a good fitting effect can be achieved. Likewise, applying the proposed model of Yeoh type leads to the same conclusion.

### 4.2. Validation of the Visco-Hyperelasticity Constitutive Model

In order to further confirm the accuracy and applicability of the proposed visco-hyperelastic constitutive model, based on the test data of cyclic tension mentioned above, fit calculations were performed directly using the Yeoh pseudo-elastic constitutive model, i.e., Equations (14) and (15), and the Ogden pseudo-elastic constitutive model, i.e., Equations (16) and (17). The fitted parameters are presented in Table 8 and Table 9.

**Figure 15:**
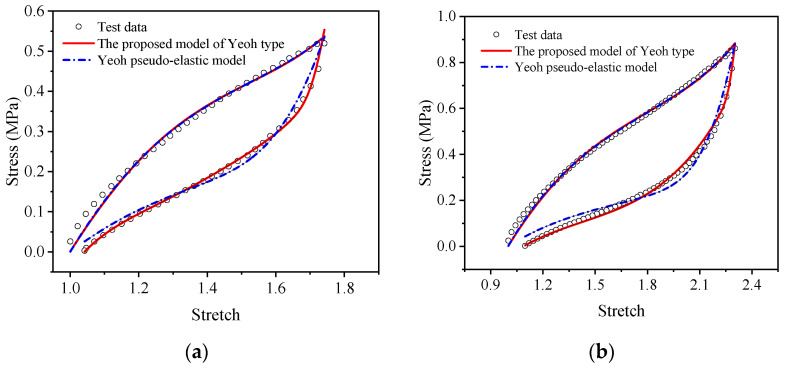
Comparison of the proposed visco-hyperelastic constitutive model of Yeoh type and the Yeoh pseudo-elastic constitutive model in the uniaxial tensile cycling tests of EPDM material: (**a**) A-type cycle; (**b**) B-type cycle.

**Figure 16:**
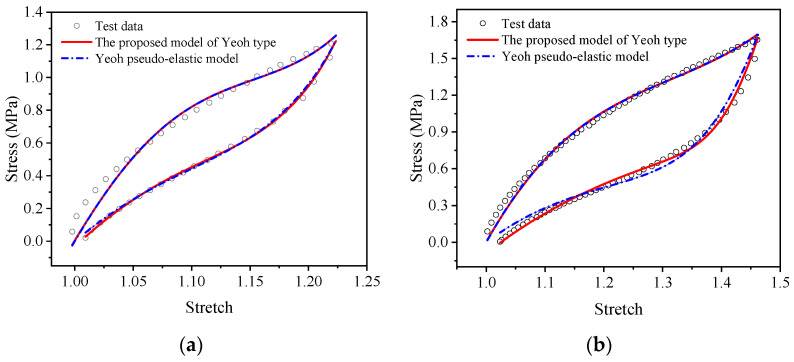
Comparison of the proposed visco-hyperelastic constitutive model of Yeoh type and the Yeoh pseudo-elastic constitutive model in the equi-biaxial tensile cycling tests of EPDM material: (**a**) A-type cycle; (**b**) B-type cycle.

Figure 15 and Figure 16 show the comparison results between the Yeoh pseudo-elastic constitutive model and the proposed visco-hyperelastic constitutive model of Yeoh type in uniaxial tensile cycling tests and equi-biaxial tensile cycle tests of EPDM rubber.

As shown in Figure 15 and Figure 16, it was found that there is almost no difference between the Yeoh pseudo-elastic model and the proposed model of Yeoh type at the loading stage, regardless of uniaxial or equal biaxial stretching. However, at the unloading stage, the Yeoh pseudo-elastic model does not match the experimental data very well, especially when the stress is close to zero, while the proposed visco-hyperelastic constitutive model of Yeoh type matches the experimental data well throughout the entire unloading process. This indicates that the proposed model is better than the Yeoh pseudo-elastic model.

**Figure 17:**
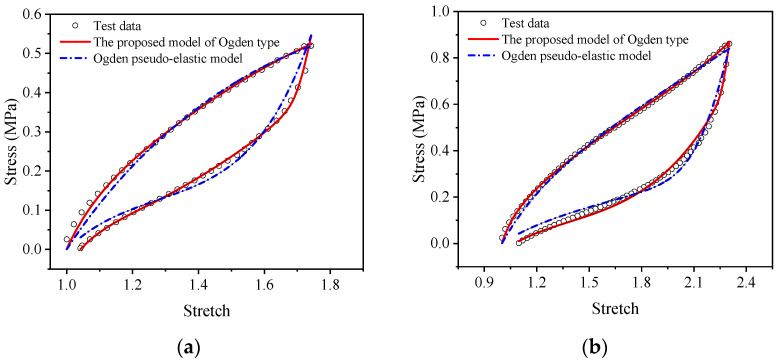
Comparison of the proposed visco-hyperelastic constitutive model of Ogden type and the Ogden pseudo-elastic constitutive model in the uniaxial tensile cycling tests of EPDM material: (**a**) A-type cycle; (**b**) B-type cycle.

**Figure 18:**
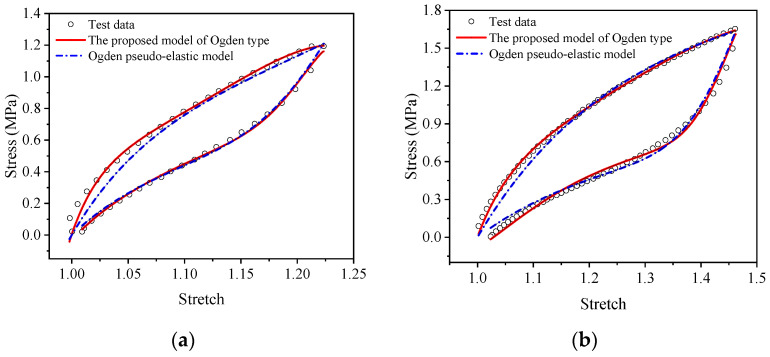
Comparison of the proposed visco-hyperelastic constitutive model of Ogden type and the Ogden pseudo-elastic constitutive model in the equi-biaxial tensile cycling tests of EPDM material: (**a**) A-type cycle; (**b**) B-type cycle.

The comparison results between the Ogden pseudo-elastic constitutive model and the proposed visco-hyperelastic constitutive model of Ogden type in the uniaxial tensile cycling tests and equi-biaxial tensile cycle tests of EPDM rubber are shown in Figure 17 and Figure 18. In the figures, we can see that the Ogden pseudo-elastic constitutive model does not approximate the EPDM rubber’s cyclic stretching test data well during the loading and unloading processes under uniaxial and equi-biaxial conditions. In contrast, the proposed model of Ogden type shows good agreement with the experimental data throughout the entire loading and unloading process and is significantly superior to the Ogden pseudo-elastic constitutive model.

Therefore, we can conclude that the proposed visco-hyperelastic constitutive model in this research can well match the cyclic test results of the EPDM material, which means it can effectively characterize the stress-softening effect in the EPDM material. The proposed model is better than the corresponding pseudo-elastic constitutive model, especially when it comes to the stress-softening unloading process.

The explanation is that the pseudo-elastic constitutive model only considers the hyper-elasticity of EPDM materials during loading and unloading and the elastic damage during unloading, but does not take the hysteresis caused by viscoelasticity into account. As a result, the load–unloading curve it describes inevitably passes through the point (1, 0) in the stress–stretch ratio coordinate system, which does not match the actual cyclic test curve of EPDM. In contrast, the proposed visco-hyperelastic constitutive model takes the hyper-elasticity, the elastic damage and the viscoelasticity into account, which is consistent with the multiplicity of polymer chain structures and molecular motions in the EPDM material. Therefore, the proposed visco-hyperelastic constitutive model is effective and reasonable. Admittedly, the proposed model has some limitations. This model is a phenomenological model and cannot accurately reflect the microstructure of the rubber-like material. In addition, the model has many parameters, causing some difficulty in parameter determination.

## 5. Conclusions

(1) The experimental results for the uniaxial and biaxial tensile tests reveal that the EPDM material presents an obvious Mullins effect during the cyclic stretching processes. Furthermore, it was found that the deformation of the EPDM material does not return to zero simultaneously with the stress, due to the viscoelasticity of EPDM material.

(2) A visco-hyperelastic constitutive model combining a hyper-elastic constitutive unit and a nonlinear viscoelastic constitutive unit based on pseudo-elasticity theory and generalized linear integral theory is proposed. The model can accurately describe the stress-softening behavior of EPDM materials. Moreover, the proposed model extends the uniaxial stress state to the biaxial stress state, which is more closely related to the actual stress state of rubber-like materials in practical engineering. This proposed model is further classified into two types: the Yeoh visco-hyperelastic constitutive model, and the Ogden visco-hyperelastic constitutive model.

(3) The proposed visco-hyperelastic constitutive model was used to fit various stress relaxation test data and cyclic tensile test data. The coefficient of determination, *R*^2^, values for the stress relaxation test data were greater than 0.986, and the *R*^2^ values for the cyclic tests were all greater than 0.971, demonstrating that the proposed model is in good agreement with the experimental data. Furthermore, the proposed visco-hyperelastic constitutive model was compared with the corresponding hyper-elastic constitutive model. The results show that the proposed model is superior to the hyper-elastic constitutive model, especially when it comes to stress-softening unloading process, which shows that it is necessary to consider the viscoelasticity of EPDM rubber in assessing cyclic tension.

(4) This work provides a two-step method for determining parameters. In the first step, we use stress relaxation tests instead of creep tests to determine viscoelastic parameters of the proposed model. The stress relaxation tests hold deformation as a constant, while the creep tests hold stress as a constant. Generally, deformation measurement is more accurate and easier to maintain stability than stress measurement. Moreover, the proposed model has the best fitting effect when the constant stretch ratio of the selected stress relaxation test is closest to the maximum stretch ratio of the cyclic test. In this work, for the A-type cyclic stretching test of EPDM rubber under uniaxial and equal biaxial conditions, the optimal constant stretch ratios are 2.08 and 1.32, respectively. For B-type cyclic stretching tests under uniaxial and equal biaxial conditions, the corresponding optimal constant stretch ratios are 2.53 and 1.51, respectively. Further research indicates that, in general, as long as the constant tensile ratio of the selected stress relaxation test is close to the maximum tensile ratio of the cyclic test, a good fitting effect can be achieved.

## Figures and Tables

**Table 1:** The composition of EPDM material.

Composition	Proportion
EPDM raw rubber	32%
Sulfurization agent	0.2%
Accelerator	0.8%
Surfactant	1.2%
Reinforcing filler	60.6%
Antioxidant	0.2%
Emollient	5%

**Table 2:** Uniaxial and equal biaxial stress relaxation test scheme of EPDM rubber.

*ST*				*ET*			
$\lambda_{0}$	Displacement (mm)	Speed (mm/s)	Time (s)	$\lambda_{0}$	Displacement (mm)	Speed (mm/s)	Time (s)
2.08	33	1	7200	1.32	25	1	7200
2.30	42	1	7200	1.41	31	1	7200
2.53	50	1	7200	1.51	38	1	7200

**Table 3:** Uniaxial and equal biaxial cyclic tensile test scheme of EPDM rubber.

Test		Load (mm)	Unload (mm)	Speed (mm/s)
*ST*	A	0–25	25–0	0.5
	B	0–45	45–0	0.5
	C	0–16	16–0	0.5
		0–32	32–0	0.5
		0–50	50–0	0.5
*ET*	A	0–20	20–0	0.5
	B	0–35	35–0	0.5
	C	0–13	13–0	0.5
		0–26	26–0	0.5
		0–39	39–0	0.5

**Table 4:** Loading and unloading strain rates in uniaxial and equi-biaxial tests of EPDM rubber.

Test		${\dot{\varepsilon }}_{L}$	${\dot{\varepsilon }}_{U}$
*ST*	A	0.0155	−0.0157
	B	0.0148	−0.0148
*ET*	A	0.0062	−0.0063
	B	0.0067	−0.0070

**Table 5:** The parameters of the viscoelastic unit of the proposed visco-hyperelastic constitutive model for uniaxial and equal biaxial cases of EPDM rubber under different stretch ratio levels.

Test	$\lambda_{0}$	$E_{\infty }$	$E_{1}$	$E_{2}$	$\tau_{1}$	$\tau_{2}$	$R^{2}$
*ST*	2.08	0.1188	0.02734	0.01911	88.58	2091.00	0.9898
	2.30	0.1052	0.02548	0.01871	90.46	2172.00	0.9906
	2.53	0.1049	0.02478	0.01894	80.67	1939.00	0.9898
*ET*	1.32	0.5642	0.1504	0.09726	81.19	2100.00	0.9875
	1.41	0.5282	0.1436	0.08825	73.28	1942.00	0.9862
	1.51	0.4490	0.1229	0.08146	74.00	2061.00	0.9873

**Table 6:** The determined parameters of the hyper-elastic unit of the proposed visco-hyperelastic constitutive model of Yeoh type for uniaxial and equal biaxial cases of EPDM rubber.

Test		$\lambda_{0}$	Load				Unload		
			$C_{10}$	$C_{20}$	$C_{30}$	$R^{2}$	r	m	$R^{2}$
*ST*	A	2.08	0.1501	−0.0811	0.02414	0.9903	1.3790	0.0082	0.9968
		2.30	0.1574	−0.0750	0.02157	0.9904	1.5110	0.0138	0.9959
		2.53	0.1578	−0.0747	0.02152	0.9904	1.4860	0.0147	0.9958
	B	2.08	0.1280	−0.0338	0.0040	0.9954	1.0980	0.0039	0.9774
		2.30	0.1372	−0.0320	0.0039	0.9956	1.2750	0.0092	0.9932
		2.53	0.1377	−0.0318	0.0039	0.9956	1.2300	0.0102	0.994
*ET*	A	1.32	0.6637	−0.9669	0.8774	0.9718	1.1360	0.0636	0.9983
		1.41	0.6857	−0.9590	0.8741	0.9718	1.1990	0.0718	0.998
		1.51	0.7327	−0.9462	0.8688	0.9717	1.3000	0.0915	0.9977
	B	1.32	0.3756	−0.1725	0.0430	0.9928	0.5832	0.0451	0.9873
		1.41	0.3984	−0.1673	0.0424	0.9929	0.6599	0.0602	0.9927
		1.51	0.4465	−0.1586	0.0412	0.9930	0.7923	0.1063	0.9936

**Table 7:** The determined parameters of the hyper-elastic unit of the proposed visco-hyperelastic constitutive model of Ogden type for uniaxial and equal biaxial cases of EPDM rubber.

Test		$\lambda_{0}$	Load							Unload		
			$\alpha_{1}$	$\alpha_{2}$	$\alpha_{3}$	$\mu_{1}$	$\mu_{2}$	$\mu_{3}$	$R^{2}$	r	m	$R^{2}$
*ST*	A	2.08	1.7030	−0.4769	0.2257	−0.2071	1.0230	−0.4958	0.9966	1.3860	0.0078	0.9978
		2.30	−0.0571	−1.4500	3.6400	−0.3666	0.7922	−0.0554	0.9935	1.5050	0.0140	0.9976
		2.53	2.8320	−0.4192	2.8600	−12.600	0.7373	12.2300	0.9930	1.4750	0.0146	0.9972
	B	2.08	−3.7080	1.2880	−2.0800	0.0137	−0.3574	0.7323	0.9988	1.0900	0.0038	0.9768
		2.30	−3.7980	0.6406	1.6240	0.6598	0.3103	−0.5153	0.9991	1.2670	0.0093	0.9921
		2.53	0.5773	−13.2600	6.5700	0.2631	0.7617	−0.4005	0.9995	1.2220	0.0103	0.9930
*ET*	A	1.32	19.2000	16.5200	−9.0790	1.5790	3.5290	−2.2560	0.9981	1.1440	0.0600	0.9977
		1.41	−6.8170	6.3960	3.8820	−1.0730	19.3600	−15.7300	0.9870	1.2100	0.0683	0.9969
		1.51	13.1900	17.1900	−8.8050	0.6069	4.6740	−2.27900	0.9879	1.3070	0.0883	0.9958
	B	1.32	−2.8810	−2.5630	5.9070	−1.7720	0.9527	1.9290	0.9977	0.5838	0.0416	0.9817
		1.41	−3.7390	5.0350	6.4350	−1.0450	−1.5490	3.9680	0.9985	0.6642	0.0596	0.9872
		1.51	3.8680	6.5250	−3.6940	−0.5164	2.9890	−1.0080	0.9985	0.7956	0.1053	0.9922

**Table 8:** The fitted parameters of the Yeoh pseudo-elastic constitutive model for uniaxial and equi-biaxial tension of EPDM rubber.

Test	Parameter	*ST*		*ET*	
		A	B	A	B
Load	$C_{10}$	0.2346	0.2200	1.0080	0.7292
	$C_{20}$	−0.0488	−0.0146	−0.8661	−0.1041
	$C_{30}$	0.0168	0.0027	0.8361	0.0340
	$R^{2}$	0.9913	0.9967	0.9712	0.9933
Unload	r	1.8410	1.5780	1.8690	1.6960
	m	0.1055	0.2115	0.2411	0.4180
	$R^{2}$	0.9858	0.9751	0.9972	0.9797

**Table 9:** The fitted parameters of the Ogden pseudo-elastic constitutive model for uniaxial and equi-biaxial tension of EPDM rubber.

Test	Parameter	*ST*		*ET*	
		A	B	A	B
Load	$\alpha_{1}$	1.6740	−1.0110	4.2510	1.1700
	$\alpha_{2}$	−1.1940	3.4500	−2.1840	1.1940
	$\alpha_{3}$	1.5880	3.3720	0.8228	−1.0920
	$\mu_{1}$	4.9170	1.6140	3.8200	−1.6850
	$\mu_{2}$	0.3152	22.1600	−0.8709	3.0400
	$\mu_{3}$	−4.7830	−23.1100	−0.9007	−0.0860
	$R^{2}$	0.9953	0.9866	0.9656	0.9859
Unload	r	1.5770	1.6610	2.2410	1.7820
	m	0.2072	0.1407	0.1847	0.3599
	$R^{2}$	0.9819	0.9755	0.9957	0.9877

## Data Availability

The data presented in this study are available on request from the corresponding author.
